# Titanium Dioxide Nanoparticles Induce Inhibitory Effects against Planktonic Cells and Biofilms of Human Oral Cavity Isolates of *Rothia mucilaginosa*, *Georgenia* sp. and *Staphylococcus saprophyticus*

**DOI:** 10.3390/pharmaceutics13101564

**Published:** 2021-09-26

**Authors:** Saher Fatima, Khursheed Ali, Bilal Ahmed, Abdulaziz A. Al Kheraif, Asad Syed, Abdallah M. Elgorban, Javed Musarrat, Jintae Lee

**Affiliations:** 1 Faculty of Agricultural Sciences, Department of Agricultural Microbiology, Aligarh Muslim University, Aligarh 202002, India; saherfatima.amu@gmail.com (S.F.); musarratj1@yahoo.com (J.M.); 2 School of Chemical Engineering, Yeungnam University, Gyeongsan 38541, Korea; jtlee@ynu.ac.kr; 3 Dental Health Department, College of Applied Medical Sciences, King Saud University, P.O. Box 10219, Riyadh 11433, Saudi Arabia; aalkhuraif@ksu.edu.sa; 4 Department of Botany and Microbiology, College of Science, King Saud University, P.O. Box 2455, Riyadh 11451, Saudi Arabia; assyed@ksu.edu.sa (A.S.); aelgorban@ksu.edu.sa (A.M.E.)

**Keywords:** oral bacteria, *Rothia mucilaginosa*, TiO_2_NPs, biofilm inhibition

## Abstract

Multi-drug resistant (MDR) bacterial cells embedded in biofilm matrices can lead to the development of chronic cariogenesis. Here, we isolated and identified three Gram-positive MDR oral cocci, (1) SJM-04, (2) SJM-38, and (3) SJM-65, and characterized them morphologically, biochemically, and by 16S rRNA gene-based phylogenetic analysis as *Georgenia* sp., *Staphylococcus saprophyticus*, and *Rothia mucilaginosa*, respectively. These three oral isolates exhibited antibiotic-resistance against nalidixic acid, tetracycline, cefuroxime, methicillin, and ceftazidime. Furthermore, these Gram positive MDR oral cocci showed significant (*p* < 0.05) variations in their biofilm forming ability under different physicochemical conditions, that is, at temperatures of 28, 30, and 42 °C, pH of 6.4, 7.4, and 8.4, and NaCl concentrations from 200 to 1000 µg/mL. Exposure of oral isolates to TiO_2_NPs (14.7 nm) significantly (*p* < 0.05) reduced planktonic cell viability and biofilm formation in a concentration-dependent manner, which was confirmed by observing biofilm architecture by scanning electron microscopy (SEM) and optical microscopy. Overall, these results have important implications for the use of tetragonal anatase phase TiO_2_NPs (size range 5–25 nm, crystalline size 13.7 nm, and spherical shape) as an oral antibiofilm agent against Gram positive cocci infections. We suggest that TiO_2_NPs pave the way for further applications in oral mouthwash formulations and antibiofilm dental coatings.

## 1. Introduction

The enormously rich and complex salivary environment of the human oral cavity provides a uniquely structured habitat for a wide variety of commensal (aerobic/anaerobic) microorganisms, and more than an estimated 700 species [1] colonize the oral cavity and form biofilms to ensure their long-term survival. Moreover, notorious biofilm persisters, including streptococci and lactobacilli, live as mutual symbionts within biofilms [2]. Furthermore, it has often been speculated that oral microbiota (bacteria, yeasts, and viruses) promote biofilm formation by producing heterogeneous extracellular polymeric substances (EPS), proteins, and nucleic acids [3]. According to Tawakoli et al. [4], the most dominant oral diseases (caries and periodontitis) are caused by bacterial adherence and subsequent biofilm formation. Multi-layered bacterial biofilm matrices play a vital role in neutralizing the antimicrobial effects of various chemical agents by acquiring drug resistance against multiple antibiotics, which is sometimes >1000-fold greater than that of planktonic cells [5,6]. The metabolism of dietary sucrose/carbohydrates creates a highly acidic microenvironment on tooth surfaces during cariogenic biofilm accumulation, and the resulting tooth surface demineralization leads to periodontal disease and tooth decay, which affect up to 60–90% of humans [7]. The problems associated with oral biofilms and their clinical management also have significant adverse economic impacts. For example, the cumulative cost of treatments for oral biofilm-related diseases has been estimated to be around USD 81 billion per annum in the United States [8].

The clinical management of biofilm-induced cariogenesis using a variety of metal nanoparticles (NPs) is now being widely explored due to their potential antimicrobial and anti-adhesive characteristics [9,10,11,12]. Amongst the metal oxides investigated, nanoscale titanium dioxide (TiO_2_) has a well-established antibacterial effect due to the production of reactive oxygen species (ROS) and cell membrane disrupting/penetrating, glutathione depleting, and toxic oxidative stress augmenting effects [13,14]. Furthermore, TiO_2_NPs are normally applied at low concentrations and are widely considered to be bio-compatible [15], though opinions differ regarding inflammation generated by cytokine release [16]. As compared with their micro/bulk-sized counterparts, TiO_2_NPs interact efficiently with a broad range of cell types (e.g., bacteria, fungi, and mammalian cells) due to their greater surface-to-volume ratios [17,18,19].

Because of its dynamic and open nature and the presence of highly complex mixes of biofilm microflora (due to host susceptibility and poor oral hygiene), the oral cavity has long been regarded as fertile ground for novel persistent biofilms. In the present study, from among ten oral isolates, we selected three Gram positive MDR cocci identified as *Georgenia* sp. (SJM-04), *S. saprophyticus* (SJM-38), and *R. mucilaginosa* (SJM-65) based on their Gram reaction, biochemical make-up, and 16S rRNA-based phylogenetic relatedness. These Gram positive MDR isolates were found resistant to nalidixic acid, tetracycline, cefuroxime, methicillin, and ceftazidime and also showed significant (*p* < 0.05) variations in biofilm formation under different experimental conditions viz., temperatures of 28, 30, or 42 °C, pH values of 6.4, 7.4, or 8.4, and NaCl concentrations of 200 to 1000 µg/mL. Exposure to TiO_2_NPs (≅14.7 nm) resulted in significant (*p* < 0.05) and concentration-dependent reductions in the viability of planktonic cells and the biofilm formation rates, and these reductions were subsequently confirmed by scanning electron and optical microscopy, respectively. Overall, these results have important implications regarding the use of TiO_2_NPs to eradicate biofilms formed by these three species.

## 2. Materials and Methods

### 2.1. Ethics Statement

Human saliva samples were collected from patients regularly visiting the Outpatient Department (OPD) of the Dr. Ziauddin Ahmad Dental College and Hospital, Aligarh Muslim University, Uttar Pradesh, India for the project CST/372 dated 14 August 2017. The patient’s consent was given before sampling. The use of saliva samples for isolation of bacteria was approved by the Internal Ethical Committee, Aligarh Muslim University, Uttar Pradesh, India.

### 2.2. Isolation and Culture Conditions

The Gram positive, oral coccoid strains, SJM-04, SJM-38, and SJM-65 were isolated from the Outpatient Department (OPD) of the Periodontics and Community Dentistry Clinic, Dr. Ziauddin Ahmad Dental College and Hospital, Aligarh Muslim University, India, by swab sampling as described by Papaioannou et al. [20]. In detail, sterile pure viscose swabs (PW043, Hi-media, Mumbai, India) were used to collect saliva samples from the floor, subgingival, and gingivae of the buccal cavities of patients. Swabs were then immediately immersed into 10 mL of sterile normal saline solution (NSS) for 30 min. Subsequently, 1000 µL samples were spread onto brain heart infusion (BHI) (Cat No. M210, Hi-media, Mumbai, India) agar plates and incubated for 24 h at 37 °C. Distinct colonies were isolated based on phenotypic characteristics (shape, size, color, margin, and colony elevation), purified, cultured, and preserved/stored in 20% glycerol at −80 °C.

### 2.3. Biochemical Characterization and Antibiotic Sensitivity Profiling of Oral Isolates

To determine the biochemical properties, IMViC, citrate, catalase, and sugar fermentation assays were performed as described in *Bergey’s Manual of Systematic Bacteriology* [21]. Resistance to 1st, 2nd, 3rd, and 4th generation antibiotic discs, that is, nalidixic acid (NA, 30 µg) and tetracycline (TE, 30 µg); norfloxacin (NX, 10 µg) and cefuroxime (CXM, 30 µg); cefotaxime (CTX, 30 µg), levofloxacin (LE, 5 µg) and ceftazidime (CAZ, 30 µg); and methicillin (MET, 5 µg) (Hi-media, Mumbai, India) was investigated and interpreted as per the CLSI guidelines (2016).

### 2.4. Assessment of Biofilm Formation at Different pH Values, Temperatures, and Salinities

Biofilm formation by the three isolates was assessed at different temperatures, pH values, and salinities. *Georgenia* sp. (SJM-04), *S. saprophyticus* (SJM-38), or *R. mucilaginosa* (SJM-65) were exposed to these various conditions in 96-wells microtiter plates. To assess the effect of pH stress, BHI broth was adjusted to pH 6.4, 7.4, or 8.4 with 0.1 M HCl or 0.1 M NaOH. For salinity tolerance, the concentration of sodium chloride (NaCl) was increased from 200 to 1000 µg/mL, and to assess the effects of temperature, microtiter plates containing pristine BHI medium were subjected to 28 °C, 37 °C, or 42 °C. Wells were inoculated with 20 µL of freshly grown test strains (≅1 × 10^6^/mL) in BHI broth. All experiments were performed in triplicate using independent bacterial colonies and data were averaged.

### 2.5. Phylogenetic Characterization of Oral Bacteria

The 16S rRNA gene amplicons of the three Gram positive oral isolates were amplified by PCR using the primers: 16S-27F (5′ to 3′AGAGTTTGATCMTGGCTCAG, M = A or C) and 16S-1492R (5′ to 3′ACGGCTACCTTGTTACGA) (Sigma-Aldrich, St. Louis, MO, USA). Qiagen DNeasy kits (Valencia, CA, USA) were used for genomic DNA extraction. For polymerase chain reaction (PCR) amplification, reaction mixtures containing 2.5U Taq polymerase (Sigma Aldrich), 100 µM of each dNTP, 0.2 µM of each primer, and 3 µL of DNA template (substrate for Taq DNA polymerase) in 50 µL of 2 mM MgCl_2_ solution were processed using a thermal cycler and the following program: 96 °C for 2 min (denaturation), followed by 30 amplification cycles of 95 °C for 15 s, 49 °C for 30 s, and 72 °C for 1 min, and a final extension at 72 °C for 1 min. PCR products were purified using the QIAquick-spin PCR Purification Kit (Qiagen, Chatsworth, CA, USA) and sequenced in a DNA sequencing facility using the BioEdit sequence alignment editor. Gene sequence homology was determined using archived 16S rRNA sequences in the GenBank server (www.ncbi.nlm.nih.gov/nucleotide) accessed on 24 January 2019, BLAST Multiple alignments of sequences, and Clustal W program. Phylogenetic trees were constructed using MEGA 6.0 and the neighbor-joining (NJ) DNA distance algorithm with bootstrap analysis (1000 replications).

### 2.6. Physicochemical Characterization of TiO_2_NPs

The physicochemical characteristics of TiO_2_NPs (Sigma-Aldrich, St. Louis, USA; product code 637254) were determined using: (i) a double beam UV-Visible spectrophotometer (UV 5704S from Electronics, India, Ltd., Panchkula, India), (ii) an X-ray diffractometer, (XRD, Rigaku Corporation, Tokyo, Japan), (iii) a transmission electron microscope, (iv) a scanning electron microscope (JSM 6510LV, SEM, Tokyo, Japan), and (v) by energy-dispersive X-ray (EDX) analyses (Oxford Instruments INCAx-sight EDX spectrometer, Concord, MA, USA). Details of the material characterization methods are provided in our earlier study [22]. Average TiO_2_NP crystalline size was determined using the Debye–Scherrer’s formula (D = 0.9λ/βcos θ; where D is crystal size, λ is X-ray wavelength, and β is the full-width at half-maximum (FWHM) of the diffraction peak).

### 2.7. TiO_2_NP-Induced Cell Growth and Biofilm Inhibition

#### 2.7.1. Dose-Dependent Anti-Planktonic Cell Activity of TiO_2_NPs

The dose-dependent antibacterial effects of TiO_2_NPs on isolated strains were determined. First, 100 µL of freshly grown (OD_600_ = 0.01) SJM-04, SJM-38, or SJM-65 strains were added to microtiter wells containing 200 µL of BHI-TiO_2_NPs (250, 500, 1000, or 2000 µg/mL) suspensions, and incubated at 37 °C in triplicate for 24 h. Untreated and treated bacterial cells were then diluted by a factor of 10^−4^ (OD_600_ = 0.01) with sterile distilled water. To determine viable cell counts, 100 µL of diluted samples (OD_600_ = 0.01) were spread on BHI agar plates and incubated at 37 °C for 24 h. The viabilities of test strains were determined by comparing the total plate counts (TPCs) of treated and untreated cells. Cells treated with or without TiO_2_NPs (250 µg/mL) were also examined for TiO_2_NP-induced morphological damage by SEM. Briefly, untreated and treated bacterial cells were spun at 3000 rpm for 5 min, fixed in glutaraldehyde (2.5%) at 4 °C for 4 h, and cell pellets were dehydrated in an ethanol series (30, 50, 70, and 90% ethanol for 15 min/step). A sample (100 µL) from each strain was mounted on a clean glass coverslip and coated with a thin layer of gold. Finally, the samples were examined under an SEM at 15 kV and 3000× [23].

#### 2.7.2. Dose-Dependent Effect of TiO_2_NPs on Biofilm Formation

The dose-dependent effects of TiO_2_NPs on biofilm formation by the three isolates were quantified by measuring crystal violet (CV) absorbance, as described by Ahmed et al. [24]. In detail, 100µL (≅1 × 10^7^ cells) of freshly grown SJM-04, SJM-38, or SJM-65 cells were added to wells containing 250, 500, 1000, or 2000 µg/mL of TiO_2_NPs in 200 µL of BHI broth per well. Cultures grown without TiO_2_NPs and sterile BHI broth alone were used as positive and negative controls, respectively. Micro-well plates were incubated at 37 °C for 24 h, and then TiO_2_NPs-BHI suspensions and loosely attached bacteria were carefully removed from the wells. Adherent biofilms on micro-well surfaces were then incubated with 200 µL of CV (1%) for 30 min, were washed with sterile PBS to remove non-absorbed CV, and air-dried. Biofilm bound CV was then solubilized with ethanol (95%) and absorbances (OD_620_) were measured using a microplate reader (Thermo Scientific Multiskan EX, REF 51118170, Shanghai, China). In a similar manner, biofilms were formed in 96-well plate for 24 h and these mature biofilms were treated with TiO_2_NPs to check the dispersal of mature biofilms by CV assay. In addition, TiO_2_NP-induced reductions in biofilm formation were also assessed by microscopy as described by Ahmed et al. [25]. Briefly, using the same conditions mentioned above, biofilms adherent to glass coverslips were washed with PBS to remove loosely attached planktonic cells and then stained with CV (1%) for 30 min. Air-dried biofilms on cover glasses were examined under an optical microscope (Olympus BX60, Model BX60F5, Olympus Optical Co. Ltd. Tokyo, Japan) equipped with a digital camera (Sony, Model no. SSC-DC-58AP, Tokyo, Japan).

### 2.8. Statistical Analyses

Multiple comparisons versus controls were performed by one-way analysis of variance (ANOVA) using the Holm–Sidak method (Sigma Plot ver. 11.0, San Jose, CA, USA). Results are presented as the means ± SDs of at least two independent experiments performed in triplicate. Statistical significance was accepted for *p* values < 0.05.

## 3. Results and Discussion

### 3.1. Isolation and Characterization of Oral Bacteria

**Figure 1:**
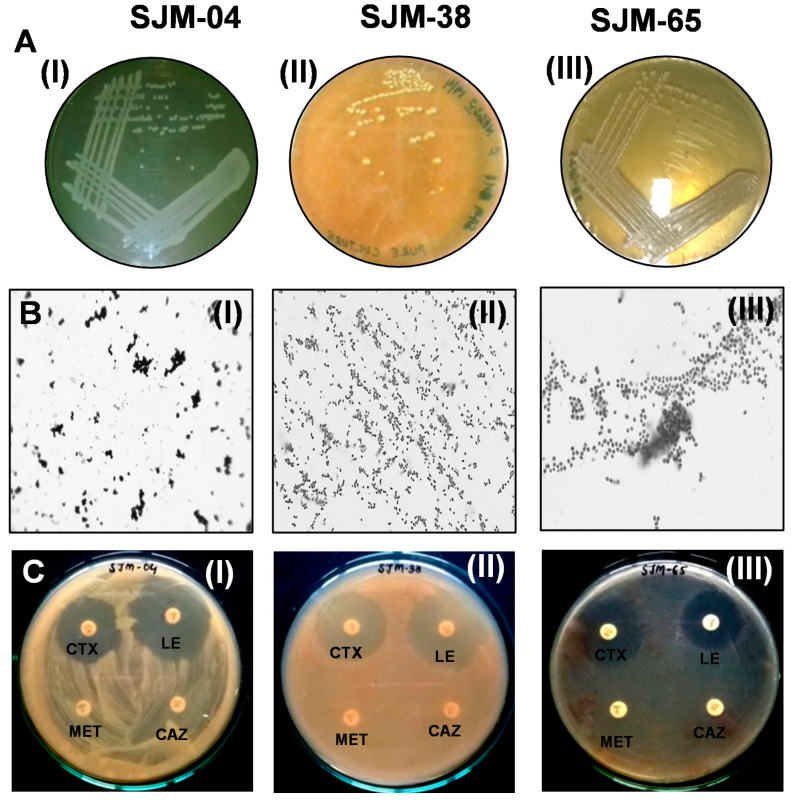
(**A**,**B**) show morphological characteristics of (**I**) SJM-04 (*Georgenia* sp.), (**II**) SJM-38 (*S. saprophyticus*), and (**III**) SJM-65 (*R. mucilaginosa*) grown onto their respective culture media. (**C**) shows representative images of the antibiotic sensitivity or resistance of (**I**) SJM-04 (*Georgenia* sp.), (**II**) SJM-65 (*S. saprophyticus*) and (**III**) SJM-38 (*R. mucilaginosa*) against multiple drugs.

The diverse oral microbiota within biofilms obtain the proteins and glycoproteins (mucins) they require to thrive from saliva [26], which is produced at a rate of 1.5–2.0 mL/min [27] and normally supports bacterial proliferation of ≅10^9^ cells/mL [28]. Therefore, we collected human saliva with sterile swabs and subsequently added sterile saline solution enriched to near-physiological saline conditions, i.e., to millimolar concentrations of NaCl and Ca^2+^ ions. Figure 1 shows the primary characteristics of the colonies of oral isolates, such as color, elevation, margin (Figure 1(AI–AIII,BI–BIII)), morphologies, and antibiotic susceptibilities (Figure 1(CI–CIII)). The Gram reactions of *Georgenia* sp. (SJM-04), *S. saprophyticus* (SJM-38), and *R. mucilaginosa* (SJM-65) confirmed their primary identities as Gram positive rods (Figure 1(BI–BIII)) and cocci. Gram positive rod *Georgenia* sp. withstood nalidixic acid (NA), methicillin (MET), and ceftazidime (CAZ). Gram positive cocci *S. saprophyticus* was resistant to MET and CAZ, whereas *R. mucilaginosa* was resistant to tetracycline (TE), cefuroxime (CXM), MET, and CAZ (Table 1). Resistance against third-generation cephalosporin reflects the presence of single-nucleotide polymorphisms (SNPs) that directly increase CAZ hydrolysis by highly conserved class A β-lactamase [29] bacterial isolates. Similarly, Higashida et al. [30] in a study on eight *S. saprophyticus* strains also showed β-lactam resistance was due to *mecA* gene-mediated resistance. Moreover, transposon mutagenesis experiments have confirmed the role of *mecA* in conferring methicillin resistance [31]. Besides presenting as urinary tract infection bacterium, *S. saprophyticus* has been isolated from a variety of other samples such as different brands of minas cheese and beach water [32].

### 3.2. Biochemical Characterizations of the Three Oral Isolates

The survival of oral communities largely relies on the nature of the salivary environment (pH, temperature, and ionic strength) and the intrinsic metabolic responses of these communities to the salivary biochemical milieu. We subjected the oral isolates to 14 different biochemical tests. The biochemical abilities of SJM-04, SJM-38, and SJM-65, to metabolize monosaccharide and disaccharide and produce citrate, cytochrome oxidase, nitrate reductase, amylase, and lipase were determined using Voges–Proskauer (VP), sucrose fermentation, citrate, catalase, nitrate reductase, starch, and lipid hydrolysis assays, respectively (Table 2). According to Kampfer et al. [33], most strains of *Georgenia* species are able to utilize glucose and sucrose, and also showed that a Gram positive coccoid *Georgenia* sp. (~1–1.5 mm) isolate with a positive oxidase reaction demonstrated aerobic metabolism. The isolate SJM-38, identified as *S. saprophyticus*, a Gram positive cocci, is commonly found in the female urinary tract [34], but has also been isolated from meat, raw milk, cheese products [35], and the marine environment in polluted and recreational waters [32,36]. Recently, Uttatree and Charoenpanicha [37] reported certain biochemical properties of *S. saprophyticus* including fermentation and oxidation of glucose and sucrose, as we detected in the current study. Additionally, *S. saprophyticus* strains exhibited the production of citrate, catalase, amylase, and lipase (Table 2). The negative reaction of VP was well supported by the literature [38]. At least four types of nitrate-reducing enzymes have been reported in oral microflora, (i) periplasmic (NAP), (ii) membrane-bound (NAR), (iii) ferredoxin-dependent assimilatory (FdNAS), and (iv) flavin-dependent assimilatory (FAD-NAS), which exhibit distinct biochemical and catalytic properties of bacterial species including *R. mucilaginosa*, *R. dentocariosa*, and *S. epidermidis* [39]. Moreover, the isolate SJM-65 *R. mucilaginosa* was found to share a positive catalase reaction and a coccoid morphology with Staphylococci species [40]. Recently, Dhital et al. [41] reported a common starch hydrolytic reaction, whereby oral isolates secrete amylolytic enzymes that convert complex starch oligomers into simpler forms.

### 3.3. Phylogenetic Identification

**Figure 2:**
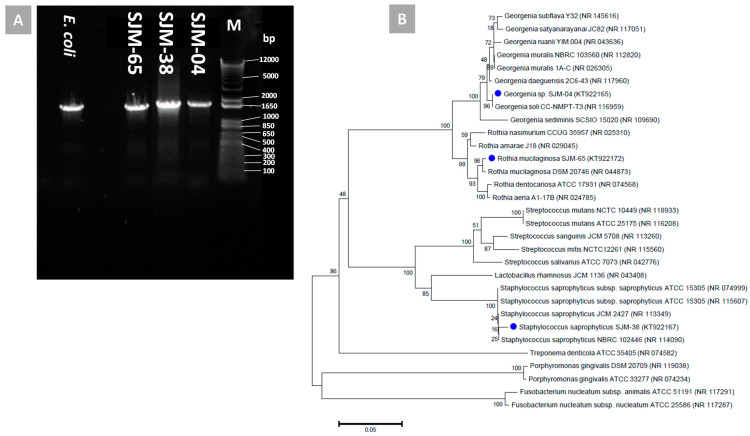
(**A**) shows an agarose gel electrophoresis result for purified 16S rDNA amplicons obtained after PCR amplification using genomic DNAs extracted from biofilm forming oral isolates as templates. (**B**) shows an unrooted neighbor-joined phylogenetic tree of closely related phylogenetic species based on 16S rRNA gene sequences of isolates SJM-04, SJM-38, and SJM-38 (marked with blue symbols). Sequences were aligned using the Clustal W sequence alignment tool in MEGA 7.0 software. The GenBank accession numbers of isolates and closely related species are presented in parenthesis. Bootstrap percentage values as obtained from 1000 replications of the data set are given at tree nodes. The scale bar represents the mean number of nucleotide substitutions per site.

Phylogenetic characterizations of the three bacterial isolates viz., SJM-04, SJM-38, and SJM-65 were carried out by analyzing 16S rRNA gene sequence homologies. PCR amplification (Figure 2A) and sequencing yielded partial nucleotide sequences of the 16S rRNA genes of SJM-04, SJM-38, and SJM-65, which were deposited in NCBI GenBank under accession nos. KT922165, KT922167, and KT922172, respectively. Phylogenetic analysis results of SJM-04, SJM-38, and SJM-65 concurred with the results of their presumptive identification as Gram positive cocci, and identified them as *Georgenia* sp. (accession no. KT922165), *S. saprophyticus* (accession no. KT922167), and *R. mucilaginosa* (accession no. KT922172), respectively. Furthermore, BLAST multiple alignments of the 16S rRNA sequence of isolate SJM-04 showed gene sequence homology with *Georgenia* sp. (accession no. KT922165) and close relatedness with the earlier recognized *Georgenia* species *Georgenia soli* strain CC-NMPT-T3 (FN356976) [33] and *G. daeguensis* strain 2C6-43 (HQ246163) [42], as shown in Figure 2B. Phylogenetic comparison of the *Staphylococcus* isolate (SJM-38) showed greatest similarity with *S. saprophyticus* strain ATCC 15305 (Figure 2B). In an identical manner, isolate SJM-65 was identified as *R. mucilaginosa* (accession no. KT922172) and showed close relatedness to all recognized species of genus Rhothia: *R. mucilaginosa* DSM 20746 and *R. dentocariosa* ATCC-17931 (Figure 2B).

### 3.4. Effects of Temperature, pH, and NaCl on Biofilm Formation

**Figure 3:**
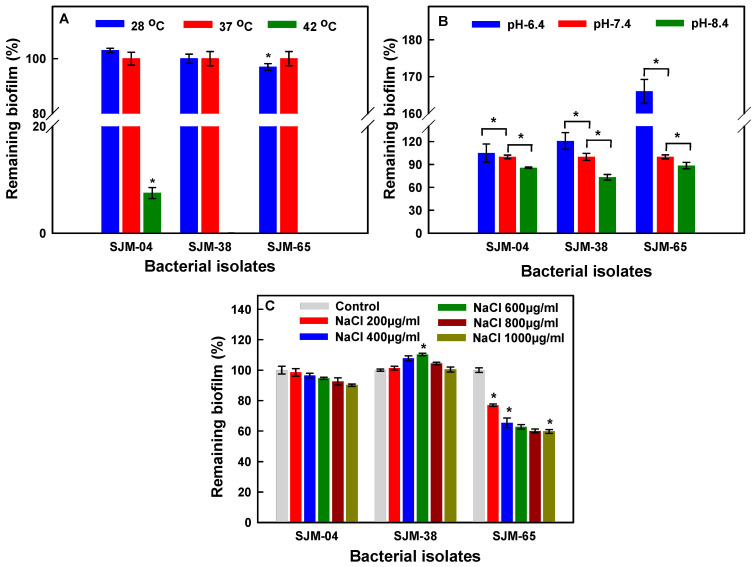
Assessment of biofilm formation as a function of temperature (**A**), pH (**B**), and NaCl concentration (**C**). ‘*’ denotes the statistical difference at *p* ≤ 0.05 between control and treated groups.

Growth patterns of bacterial cells proportionally affect the growths of biofilms, which are largely composed of non-replicating persister cells in an extracellular polysaccharide (EPS) matrix [43,44]. Unlike free planktonic cells, biofilm embedded/phenotypically altered cells become acclimatized to withstand microenvironmental stresses such as temperature, pH, and ionic strength changes. Hence, the present study primarily ascertains the optimal biofilm formation by modulating the physiochemical growth conditions for *Georgenia* sp. (SJM-04), *S. saprophyticus* (SJM-38), and *R. mucilaginosa* (SJM-65). Our results demonstrated (Figure 3A) that a lower temperature (28 °C) had a negligible effect on biofilm formation by bacterial strains as compared with the control temperature (37 °C). However, temperature elevation to 42 °C significantly limited biofilm adherence to 7.2 ± 1.0% (*p* < 0.05) for strain *Georgenia* sp. (SJM-04), and *S. saprophyticus* (SJM-38) and *R. mucilaginosa* (SJM-65) could not survive this temperature. It is widely accepted that the optimum temperature is directly related to the metabolic activities of microbial enzymes, and thus, nutrient metabolism [45] and biofilm formation [46].

In the present study, an increase or decrease in one pH unit from the control level (pH—7.4) significantly (*p* < 0.05) affected the interaction between isolates *Georgenia* sp. (SJM-04), *S. saprophyticus* (SJM-38), and *R. mucilaginosa* (SJM-65) and glass surfaces (Figure 3B). At pH 6.4, significant (*p* < 0.05) increases in biofilm formation were observed for *Georgenia* sp. (SJM-04), *S. saprophyticus* (SJM-38), and *R. mucilaginosa* (SJM-65) strains by 105.2%, 120.7%, and 166%, respectively, as compared to 100% for controls at pH 7.4). Conversely, an increase in pH to 8.4 caused significant (*p* < 0.05) reductions in biofilm to 85.77 ± 0.80, 73.26 ± 3.6, and 88.57 ± 4.2%, respectively (Figure 3B). Thus, our results indicate that a slight change in external pH can overwhelm the cellular processes that support oral bacterial biofilms, which may include the synthesis of proteins [47] and polysaccharides [48] and the membrane electrochemical gradient [49]. Earlier studies on acyl-homoserine lactone (AHL) production in quorum sensing (QS) systems of marine bacteria demonstrated salinity dependence [50], and suggested that salinity is a significant factor for QS [51]. Therefore, we also investigated biofilm growth in the presence of different concentrations (200, 400, 600, 800, and 1000 µg/mL) of NaCl. Results demonstrated a dose-dependent decrease in biofilm formation from 98.5 ± 2.5% to 90.0 ± 8.7% and from 76.9 ± 2.8% to 59.7 ± 1.2% on increasing the NaCl concentration from 200–1000 µg/mL for *Georgenia* sp. (SJM-04) and *R. mucilaginosa* (SJM-65), respectively (Figure 3C). Interestingly, under identical conditions, biofilm formation by *S. saprophyticus* (SJM-38) slightly increased on increasing the NaCl (200–1000 µg/mL) concentration 101.3 ± 1.2%, 107.7 ± 1.6%, 110.3 ± 0.7%, 104.4 ± 0.7% and 100.4 ± 1.6%, respectively (Figure 3C). According to Moretro et al. [52], NaCl and glucose stimulate adherence and increase the stability of biofilms formed by *Staphylococci* genus due to the presence of the icaA gene, which is positively correlated with strong biofilm formation. Recently, Xu et al. [53] also reported that NaCl significantly increased biofilm formation by *S. aureus* in an concentration-dependent manner

### 3.5. Physicochemical Characteristics of TiO_2_NPs

**Figure 4:**
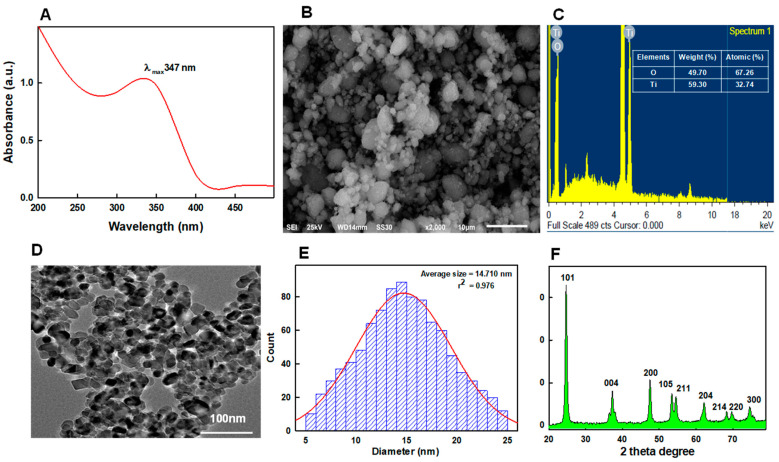
UV-Vis absorption spectrum of TiO_2_NPs showing the characteristic SPR peak at 347 nm (**A**). (**B**,**C**) show the SEM image and energy dispersive X-ray analysis of TiO_2_NPs, respectively. (**D**) shows a representative TEM micrograph of TiO_2_NPs and (**E**) shows the TiO_2_NP size distribution. (**F**) shows the X-ray diffraction pattern of TiO_2_NPs.

Surface plasmon resonance (SPR) happens due to the collective oscillations of electrons at the resonant frequency of metal NPs and results in absorption in the UV-Visible region [13]. In the present study, the appearance of a sharp peak at an absorption wavelength (λ_max_) of 347 nm in the UV-Vis absorption spectrum is likely to be due to localized SPR of TiO_2_NPs in aqueous suspension (Figure 4A). Furthermore, SPR frequencies of NPs are considered to be directly correlated with nanoparticle size, shape, and crystalline nature [54]. Therefore, we analyzed the morphology and composition and determined the size and crystallinity of TiO_2_NPs. SEM analysis showed NPs had pleomorphic shapes, though the majority were spherical (Figure 4B). The EDX spectrum of TiO_2_NPs revealed the presence of titanium (Ti) and oxygen (O) at elemental compositions of 32.74% and 67.26%, respectively (Figure 4C). TEM results showed TiO_2_NPs shapes included spherical, oval, and hexagonal particles (Figure 4D) with sizes ranging from 5–25 nm (average diameter 14.7 nm) (Figure 4E). Furthermore, the XRD pattern of TiO_2_NPs (Figure 4F), obtained by using cell parameters: a-3.8101 Å, b-3.8101 Å, and c-9.3632 Å; α = β = γ = 90° and centered tetragonal phase, showed the anatase phase TiO_2_-NPs (JCPDS 21–1272) and peaks at 2θ values of 24.6°, 37.2°, 47.5°, 53.4°, 54.6°, and 62.2° corresponding to (101), (004), (200), (1050), (211), and (204) HKL miller indices, respectively. Average size by XRD was determined to be 13.7 nm based on full-width at half-maximum (FWHM) of the 101 reflection peak, which matched well with that of the TEM size.

**Figure 5:**
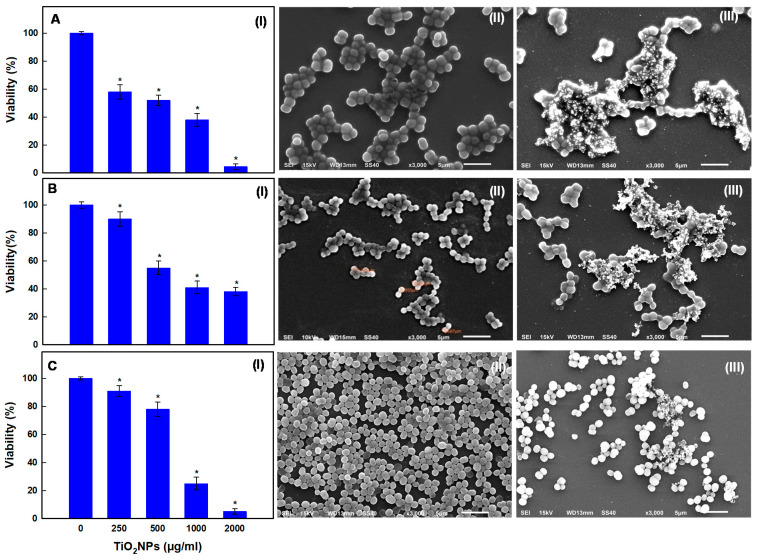
Reductions in the percentage viability of Gram positive SJM-04 (**AI**), SJM-38 (**BI**), and SJM-65 (**CI**) treated with 250, 500, 1000, or 2000μg/mL concentrations of TiO_2_NPs for 24h. SEM micrographs showing morphological changes in SJM-04 (**AIII**), SJM-38 (**BIII**), and SJM-65 (**CIII**) cells after treatment with TiO_2_NPs at 250μg/mL vs. untreated control cells (**AII**–**CII**). ‘*’ denotes the statistical difference at *p* ≤ 0.05 between control and treated groups.

The dose-dependent antibacterial effect of TiO_2_NPs (250–2000 µg/mL) on oral bacterial strains: *Georgenia* sp. (SJM-04), *S. saprophyticus* (SJM-38), and *R. mucilaginosa* (SJM-65) exhibited significant growth inhibition over 24 h (Figure 5). We found that treatment with TiO_2_NPs at 250, 500, 1000, and 2000 µg/mL for 24 h reduced the viability of *Georgenia* sp. (SJM-04), *S. saprophyticus* (SJM-38), and *R. mucilaginosa* (SJM-65) to 58.0 ± 5.2, 52.0 ± 3.8, 38.0 ± 4.5 and 4.5 ± 2.0% (Figure 5(AI)); 91.0 ± 3.8, 78.0 ± 5.2, 25.0 ± 4.5 and 5.0 ± 2.0% (Figure 5(BI)); and, 90.0 ± 5.2, 55.0 ± 4.8, 41.0 ± 4.5 and 38.0 ± 3.0% (Figure 5(CI)), respectively. The mechanisms responsible for the antibacterial activities of various metal-oxide NPs are unclear, though it is believed that the presence of dissolved metal ions on surfaces of NPs and/or NP-induced oxidative stress are involved [14]. Specifically, in the case of anatase TiO_2_NPs, the photocatalytic activity of TiO_2_ in aqueous environments results in the release of hydroxyl radicals (OH•) and the subsequent formation of superoxide radicals (O_2_^−^) [55]. Therefore, it could be argued that ROS may attack polyunsaturated phospholipids in bacteria and cause DNA damage [23,25]. Additionally, we treated *Georgenia* sp. (Figure 5(AIII)), *S. saprophyticus* (Figure 5(BIII)), and *R. mucilaginosa* (Figure 5(CIII)) with TiO_2_NPs at 250 µg/mL and examined their effects by SEM. We observed that TiO_2_NP–bacteria interactions caused morphological changes such as shrinkage and cell membrane damage, possibly because NPs penetrated bacterial membranes and compromised cell membrane permeability [56].

### 3.6. Dose-Dependent Effects of TiO_2_NPs on Biofilm Formation

**Figure 6:**
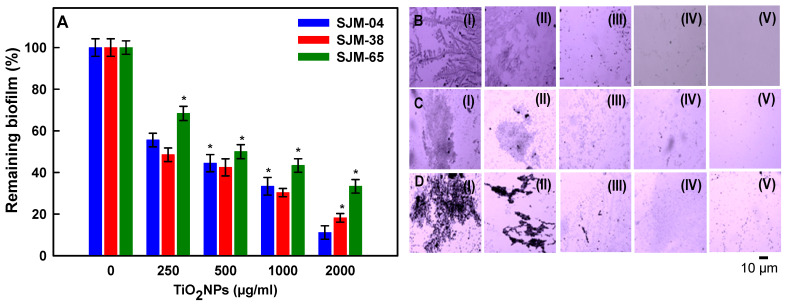
Reductions in biofilm formation after treatment with TiO_2_NPs at 250, 500, 1000, or 2000 μg/mL for 24 h (Panel **A**). Error bars denote standard deviations of triplicate samples (**A**). Microscopic images showing reduced biofilm formation by SJM-04 (**B**), SJM-38 (**C**), and SJM-65 (**D**) after treatments with TiO_2_NPs at 0 (**I**), 250 (**II**), 500 (**III**), 1000 (**IV**), or 2000μg/mL (**V**). ‘*’ denotes the statistical difference at *p* ≤ 0.05 between control and treated groups.

The effects of TiO_2_NPs concentration (250–2000 µg/mL) on the adherence of the biofilms produced by the three oral strains were also examined. The biofilms produced by various bacterial species play decisive roles in the way they respond to their immediate surroundings. Treatment of *Georgenia* sp., *S. saprophyticus*, and *R. mucilaginosa* with TiO_2_NPs at 250, 500, 1000, or 2000 µg/mL for 24 h significantly reduced biofilm adhesion on glass surfaces to 55.5 ± 3.2, 46.4 ± 4.1, 35.3 ± 4.2, and 13.1 ± 3.2%; 48.4 ± 3.2, 42.4 ± 4.1, 30.3 ± 1.9, and 18.1 ± 2.0%; and 68.3 ± 3.4, 50.2 ±3.3, 43.3 ± 3.2, and 33.3 ± 3.2%, respectively (Figure 6A). Taken together, these results show that TiO_2_NPs reduced biofilm adherence in a concentration-dependent manner. Recently, Sodagar et al. [57] reported that treatment with 5% TiO_2_NPs significantly inhibited biofilm formation by the Gram positive oral bacteria *S. mutans* and *S. sanguinis*. Additionally, the micrographs presented in Figure 6B–D show than TiO_2_NPs reduced biofilm formation by *Georgenia* sp. (Figure 6(BII–BV)), *S. saprophyticus* (Figure 6(CII–CV)), and *R. Mucilaginosa* (Figure 6(DII–DV)) in a concentration-dependent manner versus untreated controls (Figure 6(BI,DI)). In assessing the reduction in mature (24 h) biofilms of the three tested strains of TiO_2_NPs by CV assay, only 1000 or 2000 µg/mL resulted in significant destruction of mature biofilms, suggesting that TiO_2_NPs are more efficient against developing biofilms at 250–500 µg/mL, but they can also destroy biofilms at higher concentrations of 1000 or 2000 µg/mL (Supplementary Materials, Figure S1).

## 4. Conclusions

The MDR Gram positive cocci *Georgenia* sp., *R. mucilaginosa*, and *S. saprophyticus* isolated from oral cavity were successfully characterized for their morphologies, biochemical characteristics, phylogenetic relatedness, and biofilm formation at various pH, temperatures and salt concentrations. Exposure of these strains to crystalline TiO_2_NPs (5> size <25 nm) significantly inhibited their planktonic cell growth and biofilm formation. Three exposure scenarios including low (250 µg/mL), moderate (500 µg/mL), and high (1000–2000 µg/mL) doses of TiO_2_NPs decreased the biofilm in a dose-dependent manner, suggesting that the concentration of TiO_2_NPs, apart from other factors, could be the main reason why they act as both an antibacterial and antibiofilm agent to the tested oral bacteria. Our results suggest that TiO_2_NPs with the following physicochemical profile: absorption λ_max_ of 347 nm, diameter 5–25 nm, average crystalline size 13.7 nm, tetragonal anatase phase, and spherical shape might be a suitable choice for treating oral biofilms, can potentially be applied in orthodontics as a potential oral hygiene alternative to conventional rinses and for the suppression of cariogenic biofilm formation. Further in vivo biofilm studies on the interaction of TiO_2_NPs with human saliva and the effect on NP’s shape, size, and metal release are warranted for preparing the most effective antibiofilm formulations.

## Figures and Tables

**Table 1:** Antibiotic sensitivity profiling of Gram positive coccoid oral bacterial isolates.

Antibiotics	Concentration (µg/disc)	Zone of Inhibition (mm) ± S.D.		
		SJM-04	SJM-38	SJM-65
Nalidixic acid (NA)	30	0 ± 0 (R)	23 ± 2 (S)	15 ± 3 (S)
Tetracycline (TE)	10	25 ± 5 (S)	27 ± 3 (S)	0 ± 0 (R)
Norfloxacin (NX)	10	30 ± 3 (S)	27 ± 4 (S)	26 ± 4 (S)
Cefuroxime (CXM)	30	18 ± 2 (S)	32 ± 3 (S)	0 ± 0 (R)
Cefotaxime (CTX)	30	26 ± 4 (S)	30 ± 4 (S)	31 ± 2 (S)
Levofloxacin (LE)	5	32 ± 3 (S)	28 ± 5 (S)	29 ± 3 (S)
Methicillin (MET)	5	0 ± 0 (R)	0 ± 0 (R)	0 ± 0 (R)
Ceftazidime (CAZ)	30	0 ± 0 (R)	0 ± 0 (R)	0 ± 0 (R)

R = resistant and S = sensitive.

**Table 2:** Biochemical characterization of Gram positive coccoid oral bacterial isolates.

Biochemical Assay	Bacterial Isolates		
	SJM-04	SJM-38	SJM-65
Indole test	_	_	_
Methyl red	_	_	_
Voges-Proskauer	+	+	_
Citrate	+	+	+
Sucrose fermentation	+	+	_
Lactose fermentation	_	_	_
Dextrose fermentation	_	+	+
Catalase	+	+	+
Oxidase	_	+	+
Nitrate reduction	+	_	+
Starch Hydrolysis	+	+	+
Lipid Hydrolysis	_	+	+
Urease	_	_	_

‘_’ and ‘+’ signs denote a negative and positive reaction, respectively.

## Supplementary Materials

The following are available online at https://www.mdpi.com/article/10.3390/pharmaceutics13101564/s1, Figure S1. Reduction in mature (24 h) biofilms of strain SJM-04, SJM-38, and SJM-65 by TiO2NPs. ‘*’ represents statistical difference at *p* ≤ 0.05.
