# Supplementary material for: Titanium Dioxide Nanoparticles Induce Inhibitory Effects against Planktonic Cells and Biofilms of Human Oral Cavity Isolates of Rothia mucilaginosa, Georgenia sp. and Staphylococcus saprophyticus
Source: Pharmaceutics. 2021 Sep 26;13(10):1564. doi: 10.3390/pharmaceutics13101564 (PMC8540964; doi:10.3390/pharmaceutics13101564)
Supplement: Supplementary file 1 [file pharmaceutics-13-01564-s001.zip › pharmaceutics-1370408-supplementary.pdf]

pharmaceutics

Supplementary Materials: Titanium Dioxide Nanoparticles Induce Inhibitory Effects against Planktonic Cells and Biofilms of Human Oral Cavity Isolates of *Rothia mucilaginosa*, *Georgenia* sp. and *Staphylococcus saprophyticus*

Saher Fatima, Khursheed Ali, Bilal Ahmed, Abdulaziz A. Al-Kheraif, Asad Syed, Abdallah M. Elgorban, Javed Musarrat and Jintae Lee

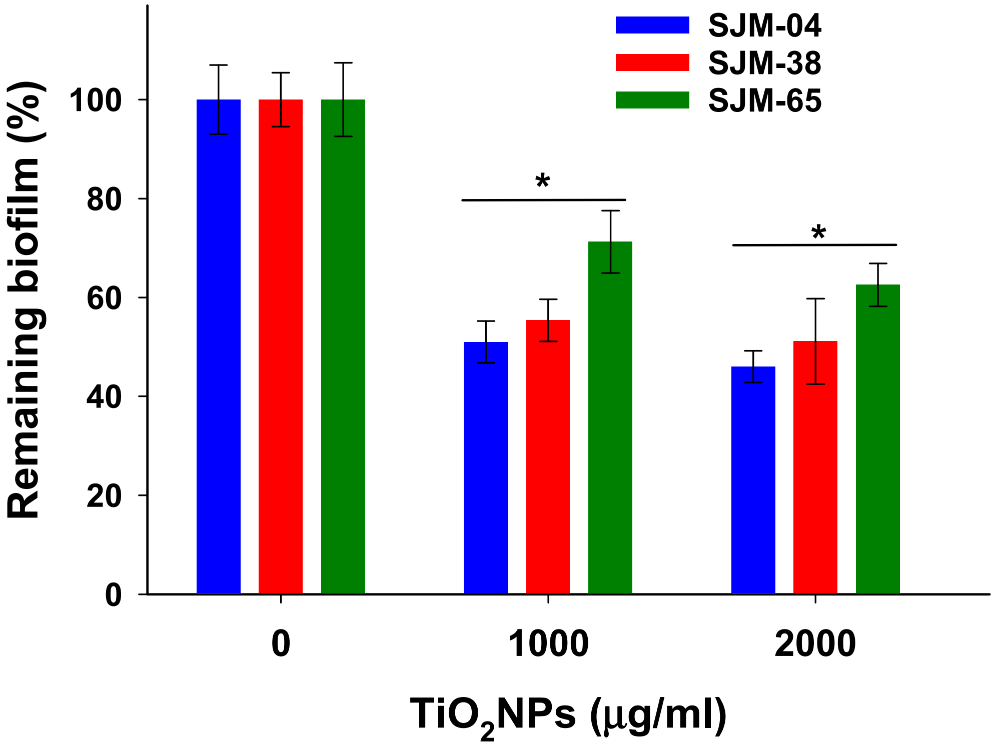

Figure S1. Reduction in mature (24 h) biofilms of strain SJM-04, SJM-38, and SJM-65 by TiO_2NPs. '*' represents statistical difference at $p \leq 0.05$.
